# Validation of the Openwater wearable optical system: cerebral hemodynamic monitoring during a breath-hold maneuver

**DOI:** 10.1117/1.NPh.11.1.015008

**Published:** 2024-03-08

**Authors:** Christopher G. Favilla, Sarah Carter, Brad Hartl, Rebecca Gitlevich, Michael T. Mullen, Arjun G. Yodh, Wesley B. Baker, Soren Konecky

**Affiliations:** a University of Pennsylvania, Department of Neurology, Philadelphia, Pennsylvania, United States; b Openwater, San Francisco, California, United States; c Temple University, Department of Neurology, Philadelphia, Pennsylvania, United States; d University of Pennsylvania, Department of Physics and Astronomy, Philadelphia, Pennsylvania, United States; e Children’s Hospital of Philadelphia, Department of Neurology, Philadelphia, Pennsylvania, United States

**Keywords:** cerebral blood flow, cerebral hemodynamics, biomedical optics, laser speckle contrast, breath-hold index

## Abstract

**Significance:**

Bedside cerebral blood flow (CBF) monitoring has the potential to inform and improve care for acute neurologic diseases, but technical challenges limit the use of existing techniques in clinical practice.

**Aim:**

Here, we validate the Openwater optical system, a novel wearable headset that uses laser speckle contrast to monitor microvascular hemodynamics.

**Approach:**

We monitored 25 healthy adults with the Openwater system and concurrent transcranial Doppler (TCD) while performing a breath-hold maneuver to increase CBF. Relative blood flow (rBF) was derived from changes in speckle contrast, and relative blood volume (rBV) was derived from changes in speckle average intensity.

**Results:**

A strong correlation was observed between beat-to-beat optical rBF and TCD-measured cerebral blood flow velocity (CBFv), R=0.79; the slope of the linear fit indicates good agreement, 0.87 (95% CI: 0.83 −0.92). Beat-to-beat rBV and CBFv were also strongly correlated, R=0.72, but as expected the two variables were not proportional; changes in rBV were smaller than CBFv changes, with linear fit slope of 0.18 (95% CI: 0.17 to 0.19). Further, strong agreement was found between rBF and CBFv waveform morphology and related metrics.

**Conclusions:**

This first *in vivo* validation of the Openwater optical system highlights its potential as a cerebral hemodynamic monitor, but additional validation is needed in disease states.

## 1 Introduction

Quantification of cerebral blood flow (CBF) at the bedside holds potential to inform and improve care for a wide range of neurologic diseases, perhaps most notably ischemic stroke in which CBF optimization is a pillar of clinical management. Unfortunately, technical limitations of existing methods for CBF quantification severely impede their clinical utility. The gold standard for non-invasive CBF imaging is $O^{15}$-positron emission tomography (PET),^1^^,^^2^ but $O^{15}\text{-}\mathrm{PET}$ is logistically complicated, expensive, and exposes the patient to ionizing radiation. Advanced MRI and CT based techniques can quantify CBF, but they provide only snapshots of data and are not suitable for serial bedside monitoring.^3^^–^^7^ Invasive tissue monitors, such as the Bowman Perfusion Monitor^®^, provide real-time physiologic data, including CBF,^8^ but they are too invasive to be practical in most clinical contexts. Thus, development and translation of a non-invasive bedside modality for CBF measurement is needed.

Transcranial Doppler (TCD) ultrasonography is widely available and is used to serially evaluate cerebral hemodynamics in clinical practice, for example, monitoring for vasospasm after subarachnoid hemorrhage.^9^^,^^10^ TCD is also employed to assess cerebrovascular reserve in both clinical and research settings by quantifying the change in CBF induced by a vasoactive stimulus, most commonly hypercapnia.^11^^,^^12^ TCD provides a measure of cerebral blood flow velocity (CBFv), rather than CBF, but this limitation is mitigated by the fact that changes in velocity are proportional to changes in flow if the arterial diameter remains unchanged.^13^ Additional limitations of TCD include the requirement of a qualified technologist, and the fact that nearly 20% of the population does not have adequate temporal acoustic windows, which may disproportionately affect females.^14^^,^^15^

Another methodology, diffuse optical imaging/monitoring, is appealing because it can circumvent some of these limitations while directly assessing tissue-level physiology. Cerebral oximetry based near-infrared spectroscopy (NIRS) is widely available and often used as a surrogate for CBF.^16^^,^^17^ However, changes in the NIRS signal may not mirror changes in CBF, e.g., if there are fluctuations in arterial oxygen saturation or cerebral metabolism,^18^^,^^19^ which is a particularly relevant limitation in cerebrovascular disease states. A qualitatively different (compared to NIRS) emerging optical modality is diffuse correlation spectroscopy (DCS). DCS quantifies the speckle intensity fluctuations of near-infrared light scattered by tissues to directly measure CBF.^20^^,^^21^ DCS has been validated against gold standard $O^{15}\text{-}\mathrm{PET}$ and other modalities,^22^^–^^26^ but signal-to-noise limitations hinder its widespread use.

In this study, we aimed to evaluate a novel, wearable optical system (Openwater, San Francisco, California) that illuminates tissue with short pulses of highly coherent laser light and leverages measurements of speckles and light intensity to continuously monitor microvascular hemodynamics. Like traditional DCS, the device quantifies the speckle intensity fluctuations of light scattered by tissues to measure CBF. The Openwater device, however, simultaneously samples millions of speckles via a speckle ensemble detection method that dramatically improves signal-to-noise ratio (SNR) compared to traditional DCS. The speckle analysis scheme, which was dubbed speckle contrast optical spectroscopy (SCOS), has been studied by several groups and typically uses a camera to measure speckle ensembles (note, this technique has also been dubbed dynamic speckle contrast analysis and dynamic speckle contrast flowmetry by early practitioners).^27^^–^^35^ A key feature of the Openwater system is its use of short pulses of very intense laser light. The use of short pulse illumination permits the dynamics of tissue located deep below the surface to be probed at short time scales while maintaining a safe low average power. One need for validation stems from this use of short pulses derived directly from within the laser system (rather than by modulation outside of the laser system). These intense laser light pulses hold potential to increase sensitivity, but the scheme is challenging to implement without introducing spectral and modal complications that can degrade contrast. The present study utilized a 36 mm source-detector distance to measure CBF at 40 Hz sampling with sufficient SNR to resolve pulsatile CBF waveforms during the cardiac cycle. We employed a breath-hold maneuver to provoke a large CBF variation in healthy volunteers to provide a means for validating the Openwater device by comparison with TCD.

## 2 Materials and Methods

### 2.1 Participants

Healthy individuals between the ages of 18 and 45 were eligible to participate. Subjects were excluded if they had a history of hypertension, type-2 diabetes, hyperlipidemia, heart failure, stroke, cerebrovascular abnormality, intracranial mass lesion, or skull defect, which could interfere with TCD monitoring at the temporal region. The study protocol was approved by the University of Pennsylvania Institutional Review Board, and all study procedures were conducted in accordance with the ethical standard of the Helsinki Declaration. All study participants provided written informed consent prior to any study procedures. The study conformed to STROBE guidelines for observational studies.

### 2.2 Optical Blood Flow Instrumentation

**Fig. 1:**
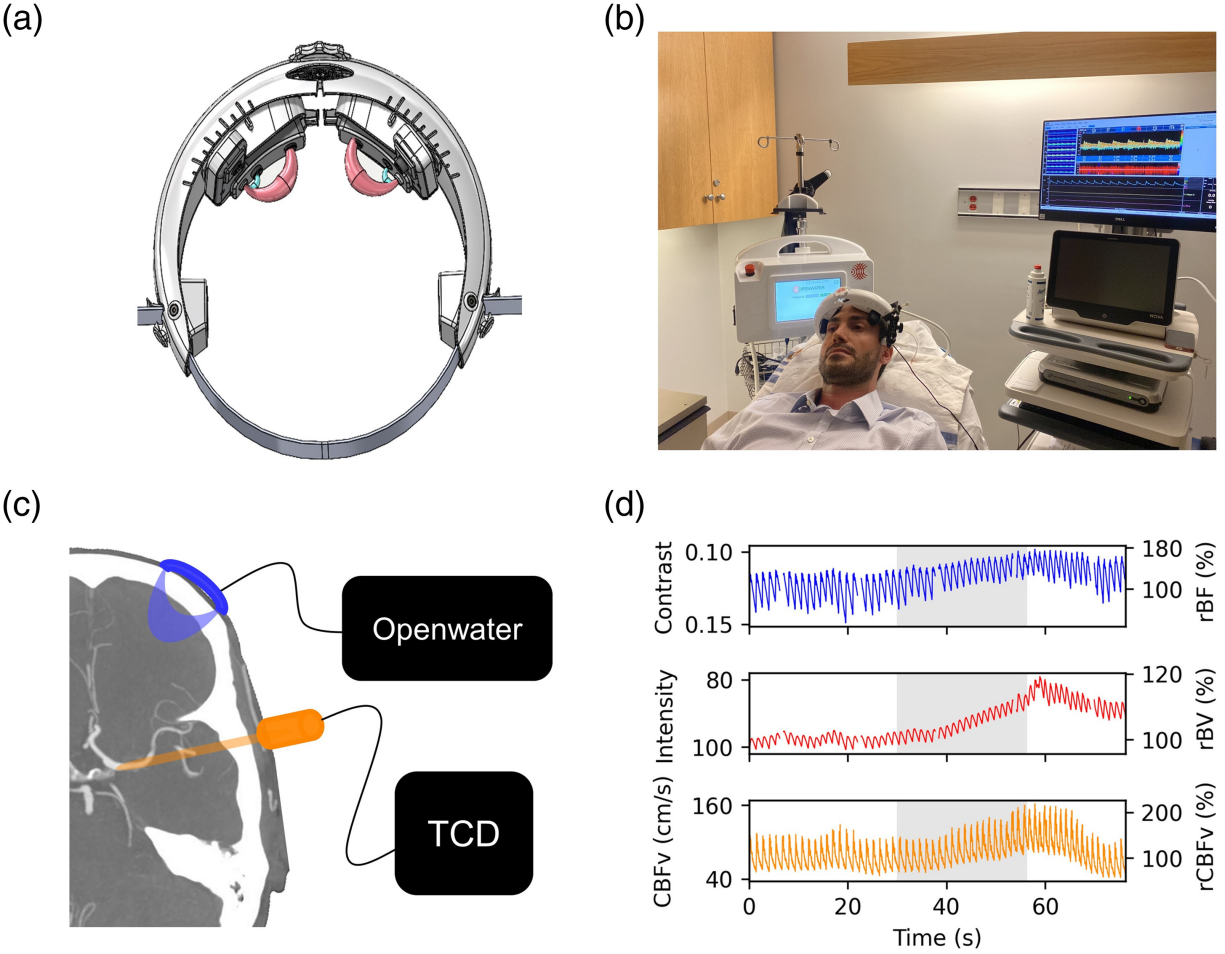
Experimental setup and raw time-series data: (a) A schematic of the Openwater headset, demonstrating the light source/detector positioning and the theoretical light path. (b) A photograph depicts the experimental set-up. The Openwater Headset is on the subject’s head, and the TCD probe is insonating the MCA. The Doppler probe is fixed to the Openwater Headset using a custom probe holder. The Openwater Headset is tethered to the console and the console is plugged into a wall outlet power source (i.e., no onboard battery). (c) The frontal lobe is probed by the Openwater Headset over the lateral aspect of the forehead. The MCA is insonated by TCD. (d) An example of time-series data demonstrates one subject’s hemodynamic data during the breath-hold maneuver. The blue line represents the speckle contrast (informative of flow). The red line represents the light intensity (informative of volume). The orange line represents CBFv as measured by TCD. The gray shaded region represents the time during which the subject was holding their breath.

The hemodynamic measurement device (Openwater; San Francisco, California) consists of a wearable headset and a console. The headset contains two modules that collect data simultaneously from both side of the head. For comparison with TCD, data from the module positioned on the left lateral aspect of the forehead, overlying the lateral frontal lobe were used (Fig. 1). In addition, the optically measured blood flow was compared between the left and right hemispheres. The modules contain a built-in optical fiber for the delivery of low average power laser light to the surface of the brain, as well as a custom camera for the measurement of light escaping from the subject. The console contains the laser, electronics, touchscreen, and computer.

The source fibers emitted 250  μs pulses of highly coherent near-infrared laser light with wavelength near the isosbestic point for hemoglobin (785 nm). An external trigger synchronized the pulsed laser with the camera. The pulses had an energy of 400  μJ and were emitted at a rate of 40 Hz. (Based on these specifications, the duty cycle was 1%, and the peak power was 1.6 W per channel.) After passing through tissue, the light pulses were collected by a custom camera module (Openwater; San Francisco, California) consisting of a 3 mm aperture and a 5-megapixel CMOS sensor (HM5530; Himax Technologies; Xinshi, Taiwan) optimized for NIR light (QE 60% at 785 nm). The aperture was positioned 36 mm from the source (Fig. S1 in the Supplemental Material), the pixel width of the sensor was 2  μm, and the sensor was recessed from the aperture by 7 mm resulting in a coherence area $\left(A_{c}=\frac{(\text{lamda}*z{)}^{2}}{A_{\text{aperture}}}\right)$ to pixel area ($A_{\text{pixel}}=4\;\mu m^{2}$) ratio, $\frac{A_{c}}{A_{\text{pixel}}}$, of 1.1, where z represents the distance between the aperture and sensor.^36^ Thus, for each camera exposure, about 5 million coherence areas (i.e., speckles) were sampled. The large aperture increased light collected while only resulting in a modest decrease in the average speckle contrast (a 30% decrease compared to an idealized scenario wherein $\frac{A_{c}}{A_{\text{pixel}}}\gg 1$). The combination of a megapixel sensor and a large collection aperture contributed to the ability of the device to make measurements at large source detector separations, which would otherwise have been overwhelmed by the read noise of the sensor. Notably, the average power was only 16 mW and was spread over an area on the tissue surface that was wide enough area such that light illumination was below the IEC-60825-1 Maximum Permissible Exposure and Class 1 limits.

Pulsing the laser light is also a critical part of the measurement method for the following reason. The laser uses a master oscillator power amplifier (MOPA) configuration. The master oscillator is run in CW mode and is fiber coupled to the power amplifier. Pulses are formed by modulating (pulsing) current to the power amplifier. The master oscillator is a volume holographic grating stabilized laser, and a tapered amplifier was used for the power amplifier. To maximize the sensitivity to CBF, it is necessary to sample the speckles on a shorter time scale than is used for single scattering laser speckle contrast imaging (LSCI). This is because the time scale of the CBF-induced decay of the temporal auto-correlation function is much shorter for multiply scattered light, which samples tissue far below the surface, as compared to a single scattered light reflected from surface/near-surface tissue (the case for LSCI). Further, to probe deeply, we need to maximize the separation between light source and detector.^21^^,^^37^^–^^39^ Unfortunately, collecting sufficient light over such a short period of time at a large source detector separation requires illumination of the subject with high-power light (i.e., several watts). If a continuous wave light source is used, then this large average power may burn the subject. The Openwater device solves this problem by using light pulses with high peak power but at a very small duty cycle. Thus, the average power is small. The long source-detector separation (compared to the 25 mm separation used in the majority of published DCS studies) increases the depth of interrogation,^39^ and when combined with the rapid measurement scheme, increases the sensitivity to CBF changes. Appendix A provides a detailed description of the instrument design, including laser specifications and supporting data.

For each image acquired on the CMOS sensor, the mean intensity I and variance $\sigma^{2}$ were computed from the digital values of the pixels on the sensor. Computations were performed by the embedded computer within the console. The variance was corrected for shot noise and read noise according, i.e., $\sigma^{2}=\sigma_{\text{raw}}^{2}-\sigma_{\text{shot}}^{2}-\sigma_{\text{read}}^{2}$. The speckle contrast (C) was then calculated for each image (without averaging multiple images): $C=\sqrt{\sigma^{2}}/I$. To account for other sources of variance including pixel non-uniformity and vignetting, an offset was subtracted from C. The offset corresponded to the contrast measured when the wavelength of the laser was modulated sufficiently rapidly such that its temporal coherence was reduced enough to eliminate the speckles. The resulting speckle contrast and mean intensity values were acquired at 40 Hz. We used linear interpolation to up-sample the (band limited) waveforms to 125 Hz to enable synchronization with TCD data (described below). Changes in blood flow and blood volume were estimated from changes in speckle contrast and mean intensity, respectively, as described below.

### 2.3 Transcranial Doppler Ultrasonography

CBFv was assessed using a Multigon Industries^®^ TCD system (Elmsford, New York). The left middle cerebral artery (MCA) was insonated via the trans-temporal window at a depth of 40 to 65 mm. The vessel was confirmed by its characteristic depth range, Doppler signal, direction, and velocity.^34^ To ensure signal stability for the duration of the monitoring period, a 2 MHz TCD probe was secured directly to the Openwater Headset using a custom clamp designed to facilitate continuous vessel insonation while minimizing motion induced artifacts or signal loss. MCA waveform (125 Hz sampling) and beat-to-beat mean CBFv were recorded and synchronized with optical data. If transient dropouts TCD occurred, these were replaced with linearly interpolated data points.

### 2.4 Cerebrovascular Reactivity Protocol

All studies were conducted in a single examination room within the neuro-diagnostic suite at the Hospital of the University of Pennsylvania. Prior to hemodynamic monitoring, subject demographics were collected on a case report form. Skin pigmentation was assessed by the Fitzgerald scale, which quantifies skin color based on a six-point scale. The study room was quiet and temperature controlled (23°C) throughout the duration of monitoring. Subjects were positioned in a hospital stretcher with the head-of-bed elevated to 45 deg. The Openwater headset [Fig. 1(a)] was placed on the participant’s head to ensure the optical probes were along the upper border of the forehead [Fig. 1(b)]. The headset size was adjusted using a built-in dial to ensure the optical probes were on the lateral margin of the forehead (while avoiding hair). The TCD probe was secured to the Openwater headset via an adjustable clamp in order to insonate the left MCA via the temporal acoustic window [Fig. 1(c)]. TCD and optical data were synchronized at the beginning of each subject’s monitoring session.

**Fig. 2:**
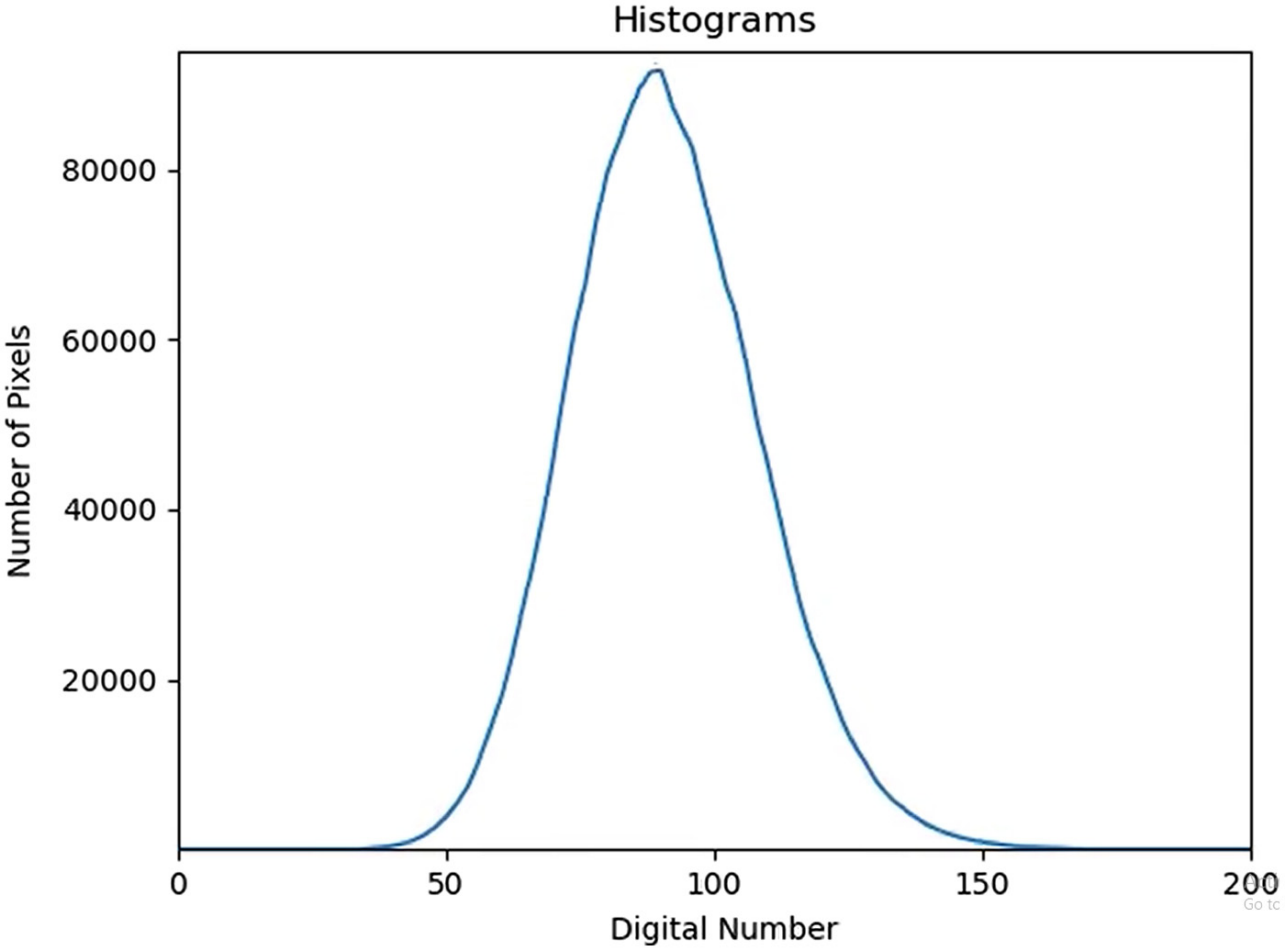
The histogram depicts how the speckle intensity varies in time during the baseline resting-state monitoring (prior to breath-hold) for a representative subject. Each time point (40 per second) exhibits a histogram of digital signals detected for each pixel across the whole sensor. The change in the histogram over time is reflective of the subject’s pulse (Video 1, MP4, 178 KB [URL: https://doi.org/10.1117/1.NPh.11.1.015008.s1]).

After confirming signal quality from both modalities, 30 s of baseline data were collected. Then, a 30-s breath-hold was performed. The breath-hold was initiated at the end of expiration to avoid pre-oxygenation and elicit a more reliable hypercapnic response. After 2 min of rest, another 30-s breath-hold was completed. The first breath-hold was used for analysis, but if the subject was unable to perform the first breath-hold or if there was signal loss with either imaging modality, then the second breath-hold was used for analysis. In the case that subjects completed both breath-holds, only the first was included in the analysis; we did not combine or average the two breath-holds because the two breath-holds may elicit different responses.^35^^–^^37^ Raw time series example data from one subject is shown in Fig. 1(d). Figure 2 shows the histogram of speckle intensity as it varies in time during the baseline monitoring prior to breath-hold, where each time point exhibits a histogram of digital signals detected for each pixel across the whole sensor.

### 2.5 Optical and TCD Data Processing

For each modality, a pulse-finding algorithm discriminated beats in the speckle contrast (optical) and CBFv (TCD) signals, from which beat-to-beat mean and pulsatility index [PI = (peak systolic value − end diastolic value)/mean] were obtained. A baseline value for each parameter was calculated as the average over the 30 s prior to initiation of the breath-hold. The relative change from baseline was calculated for each beat-to-beat value thereafter (i.e., in this way, changes from baseline were effectively normalized to facilitate inter-modality comparison):

$$\mathrm{rBF}=\text{rContrast}(t)=1-\frac{C(t)-C_{\text{baseline}}}{C_{\text{baseline}}},$$

$$\mathrm{rBV}=\text{rIntensity}(t)=1-\frac{I(t)-I_{\text{baseline}}}{I_{\text{baseline}}},$$

$$\mathrm{rCBFv}(t)=1+\frac{\mathrm{CBFv}(t)-{\mathrm{CBFv}}_{\text{baseline}}}{{\mathrm{CBFv}}_{\text{baseline}}},$$

$${\mathrm{rPI}}_{\text{Contrast}}(t)=1-\frac{{\mathrm{PI}}_{\text{Contrast}}(t)-{\mathrm{PI}}_{\text{Contrast}\_\text{Baseline}}}{{\mathrm{PI}}_{\text{Contrast}\_\text{Baseline}}},$$

$${\mathrm{rPI}}_{\text{Intensity}}(t)=1-\frac{{\mathrm{PI}}_{\text{Intensity}}(t)-{\mathrm{PI}}_{\text{Intensity}\_\text{Baseline}}}{{\mathrm{PI}}_{\text{Intensity}\_\text{Baseline}}},$$

$${\mathrm{rPI}}_{\mathrm{CBFv}}(t)=1+\frac{{\mathrm{PI}}_{\mathrm{CBFv}}(t)-{\mathrm{PI}}_{\mathrm{CBFv}\_\text{Baseline}}}{{\mathrm{PI}}_{\mathrm{CBFv}\_\text{Baseline}}}.$$

Note, we compute relative blood flow (rBF) and relative blood volume (rBV) from the fractional changes in contrast and intensity, respectively, during the monitoring session (e.g., a 20% increase in contrast reflects a 20% decrease in blood flow; a 20% increase in intensity reflects a 20% decrease in blood volume). Several models have been proposed for quantifying static and dynamic optical properties in tissue, and often the blood flow is calculated as $\frac{1}{C^{2}}$ based on an exponential model of the autocorrelation decay.^27^^,^^40^ The equations employed here are not reliant on previously described models and thus are not subject to the associated assumptions. The definition of rBF was selected as the simplest equation with the correct general behavior for small changes in blood flow. The linear model also has the advantage that it is robust in the presence of experimental noise. The noise in rBF is simply equal to the relative noise of the measured contrast (i.e., $\sigma_{\mathrm{rBF}}=\sigma_{C}/C_{\text{baseline}}$). Had we used the exponential model $\mathrm{rBF}=1+\frac{\mathrm{BF}(t)-{\mathrm{BF}}_{\text{baseline}}}{{\mathrm{BF}}_{\text{baseline}}}=\frac{\mathrm{BF}(t)}{{\mathrm{BF}}_{\text{baseline}}}=\frac{C_{\text{baseline}}^{2}}{C^{2}(t)}$, then $\sigma_{\mathrm{rBF}}=2\sigma_{C}/C(t)$). Thus, the noise in the exponential model is twice as large and may negatively impact the ability to detect subtle waveform features.

Cerebrovascular reactivity was quantified by breath-hold index (BHI) and time to maximum effect (i.e., seconds from breath-hold initiation to the maximal value for each modality). The BHI was calculated as follows:^41^^,^^42^

$$\mathrm{BHI}=\frac{\left(\text{Maximum value}-\text{Baseline value})/(\text{Baseline value}\right)}{\text{Breath-hold duration}(\text{seconds})}\times 100.$$

**Fig. 3:**
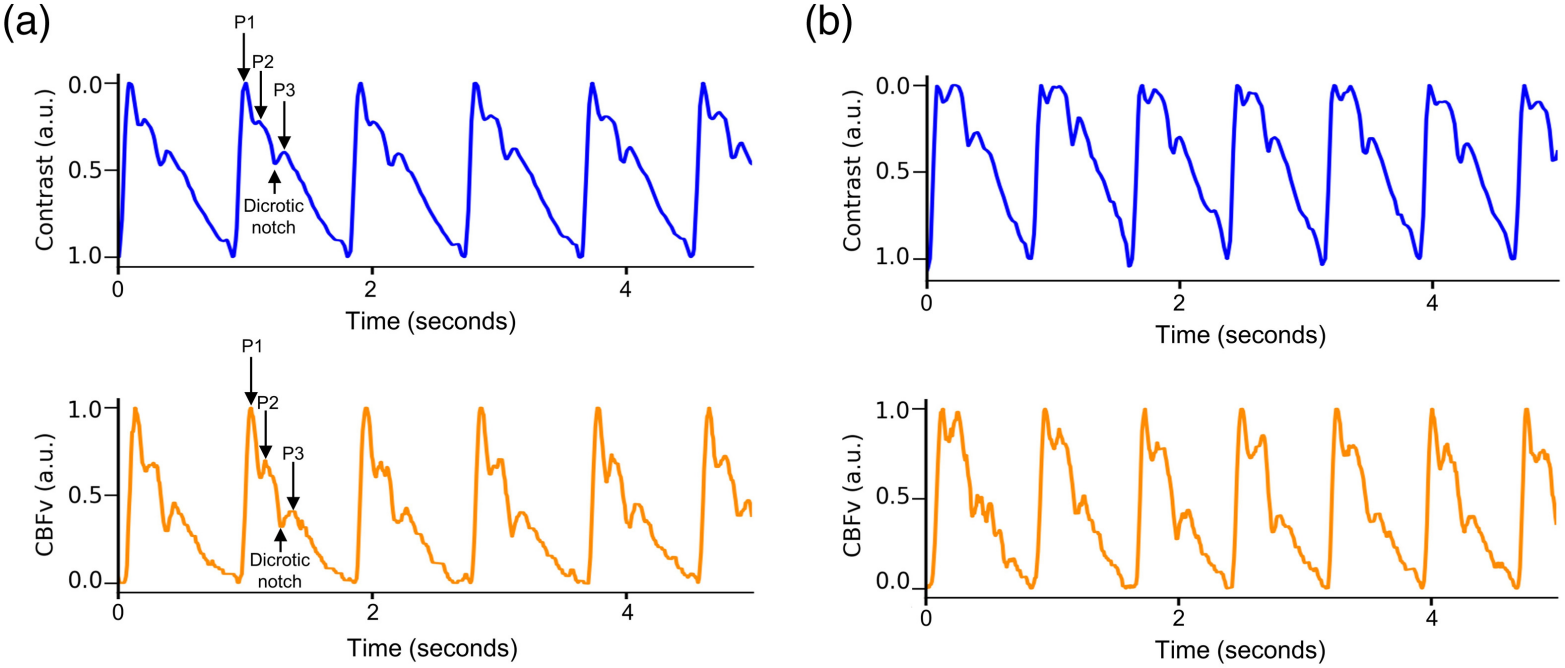
Waveform morphology before and after breath-hold: Representative raw waveform data are depicted from a single subject. All waveforms amplitudes are normalized (i.e., setting the y-axis scale from 0 to 1). (a) Prior to the initiation of the breath-hold, 5 s of data is depicted with both modalities. The dicrotic notch and three peaks are identified (P1, P2, P3). (b) At the end of the breath-hold, a change in waveform morphology, in particular an increase in the relative amplitude of P2, can be appreciated with both modalities. Again, 5 s of data are depicted. CBFv indicates cerebral blood flow velocity.

Waveform morphology was evaluated before and after the breath-hold to facilitate comparison between speckle contrast-derived blood flow waveform and the TCD-derived CBFv waveform (Fig. 3). Each pulse was normalized such that peak systolic and end diastolic values were 1 and 0, respectively. Pulses were averaged during 30 s of baseline data and separately averaged during the 10 s window centered at the time of maximum effect after breath-hold initiation, selected at the time of peak effect post-breath-hold. From these averaged pulses, a peak detection algorithm identified the dicrotic notch and three peaks: (1) P1 represents ejection of blood from the left ventricle, (2) P2 represents the pulse wave reflected by the closing aortic valve, and (3) P3 represents the diastolic flow. The augmentation index (AIx), calculated as the ratio of the amplitude of P2 to P1, provides a measure of cerebrovascular stiffness.^43^^,^^44^ AIx was calculated based on optical blood flow (rBF-AIx) and TCD (CBFv-AIx) during baseline and hypercapnia (i.e., at the end of the breath-hold). Peak finding was reviewed independently by two study team members and manually corrected if necessary; notably in the pulses where three distinct peaks were not easily discriminated.

### 2.6 Statistical Analysis

Summary statistics were presented using means and standard deviations for continuous variables, medians, and interquartile ranges for ordinal or non-parametric variables and proportions for categorical variables. After normalizing values to the baseline period, we used correlation, mixed-effects linear regression, and Bland–Altman analyses to investigate agreement on a beat-to-beat basis between: (a) mean rBF versus mean CBFv and (b) mean rBV versus mean CBFv. The Pearson R was also calculated per subject. R is bounded by −1 to 1 and not expected to be normally distributed, so the average and standard deviation of R were transformed using Fisher’s transformation (F=arctanh(R), where arctanh is the hyperbolic arctangent). The resulting values were then transformed back to correlation space via the hyperbolic tangent to report summary statistics.^45^ We used Pearson’s correlation and linear regression to investigate the agreement between the optical and TCD measurements of BHI and time to maximum effect. The timing of the three peaks (P1, P2, P3) and dichrotic notch were evaluated by correlation and linear regression in comparing the optical and TCD waveforms. The pre- to post-breath-hold measured change in PI and AIx were correlated between the two modalities. In addition, the beat-to-beat optical signals (rBF and rBV) were compared between the left and right hemispheres. The data that support the reported findings are available from the corresponding author upon reasonable request.

## 3 Results

Of the 25 subjects who completed the monitoring protocol, 2 were excluded due to poor TCD data quality, and 23 were included in the final analysis. The first breath-hold was sufficient for analysis in 21 subjects, but one subject did not correctly hold their breath on the first attempt, so the second breath-hold was analyzed for this subject. The protocol was well tolerated without any adverse events. No subjects reported headset overheating. The mean participant age was 35 years (±11). 61% of the participants were female, and the median Fitzpatrick scale of skin pigmentation was 2 (IQR: 1 to 2).

**Fig. 4:**
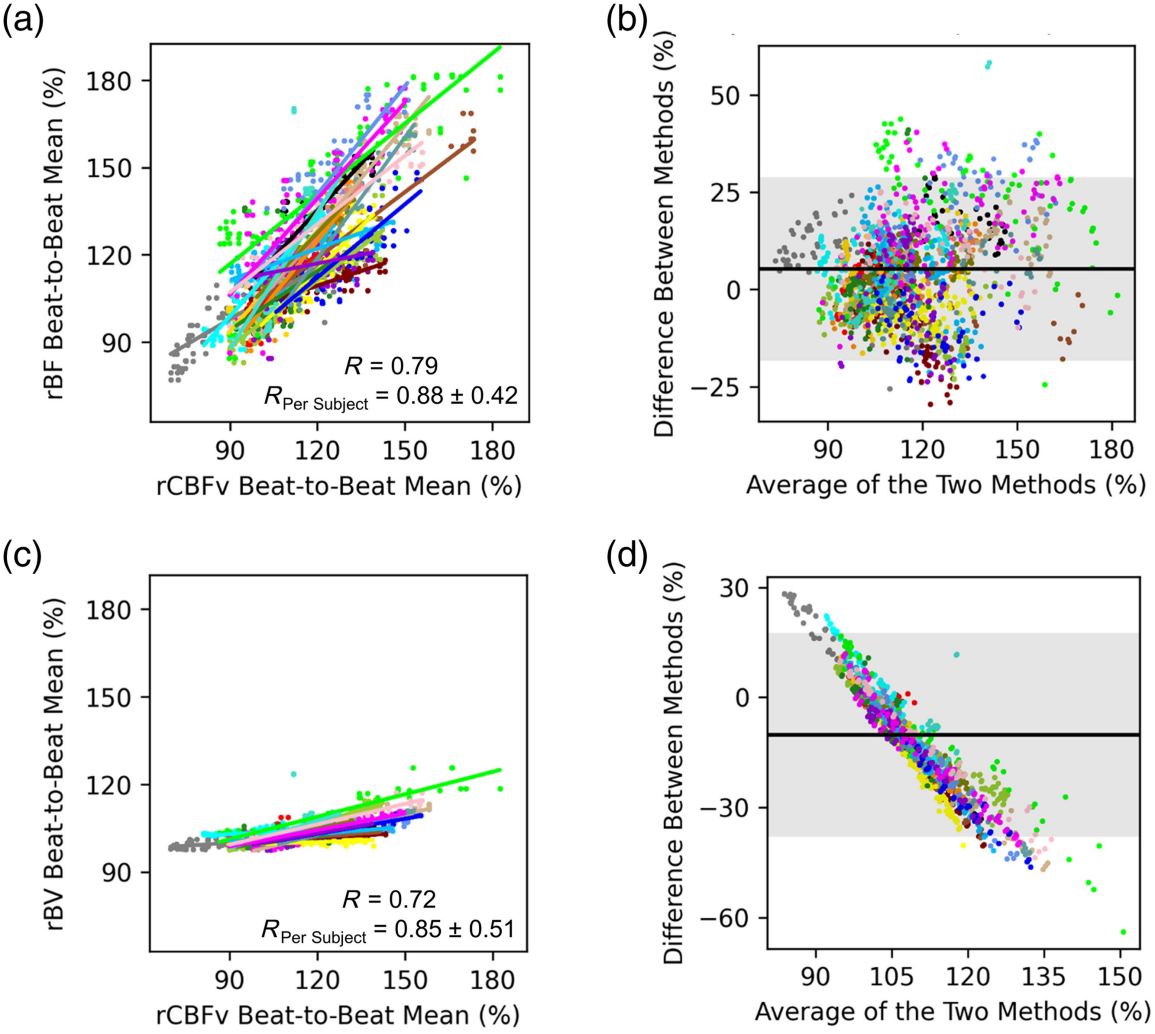
Comparing optical and TCD beat-to-beat monitoring: All data are normalized to the 30-s period preceding the breath-hold. Beat-to-beat mean values are calculated for each metric from the start of the breath-hold through 5 s after the completion of the breath-hold. Each color represents a different subject. (a) A scatterplot depicts the beat-to-beat mean rCBFv (x-axis) and the beat-to-beat mean rBF (y-axis). The overall correlation coefficient is 0.79. The average correlation coefficient (when calculated for each subject individually) is 0.88 (±0.42). The slope of the mixed-effects linear model is 0.87 (95% CI: 0.83 to 0.92). (b) A Bland–Altman plot indicates beat-to-beat mean rCBFv is on average 5% smaller than beat-to-beat mean rBF. The gray shaded region represents the 95% confidence interval for agreement. (c) A scatterplot depicts the beat-to-beat mean rCBFv (x-axis) and the beat-to-beat mean rBV (y-axis). The overall correlation coefficient is 0.72. The average correlation coefficient (when calculated for each subject individually) is 0.85 (±0.51). The slope of the mixed-effects linear model is 0.18 (95% CI: 0.17 to 0.19), which indicates that changes in rBV are smaller than changes in rCBFv. (d) A Bland–Altman plot indicates rCBFv is on average 10% larger than rBV. The gray shaded region represents the 95% confidence interval for agreement. A negative trend is evident and indicates that as the average value increases, the difference between CBFv and rBV increases. TCD indicates transcranial Doppler. rCBFv indicates TCD measured relative cerebral blood flow velocity. rBF indicates optically measured relative blood flow. rBV indicates optically measured relative blood volume.

The optical and TCD measurements of mean beat-to-beat rBF and rCBFv, respectively, demonstrated good agreement as was evidenced by a strong correlation (overall R=0.79, R per subject=0.88±0.42) and a slope of 0.87 (95% CI: 0.83 to 0.92) in the mixed effects model [Fig. 4(a)]. Based on a Bland–Altman analysis, the mean difference between the two modalities was 5%, and the vast majority of the beat-to-beat values were within the 95% confidence interval for agreement [Fig. 4(b)]. Of additional interest was the potential agreement between beat-to-beat optical blood volume (rBV) and TCD-measured CBFv [Fig. 4(c)]. The correlation was strong (overall R=0.72, R per subject=0.85±0.51), but changes in rBV were expectedly smaller than changes in rCBFv as evidenced by a slope of 0.18 (95% CI: 0.17 to 0.19) in the mixed effects model [Fig. 4(d)]. The Bland–Altman analysis indicated a mean difference between the two modalities of 10%, and there was a negative trend in the Bland-Altman plot because changes in rCBFv and rBV were not proportional. Strong agreement between optical parameters collected from the left and right hemispheres was also observed (Fig. S2 in the Supplemental Material). For example, there was a very strong correlation between the beat-to-beat mean rBF from the right and left probes (overall R=0.92; R per subject=0.96±0.04), and there was also a very strong correlation between the beat-to-beat mean rBV from the left and right probes (overall R=0.82; R per subject=0.85±0.19).

**Fig. 5:**
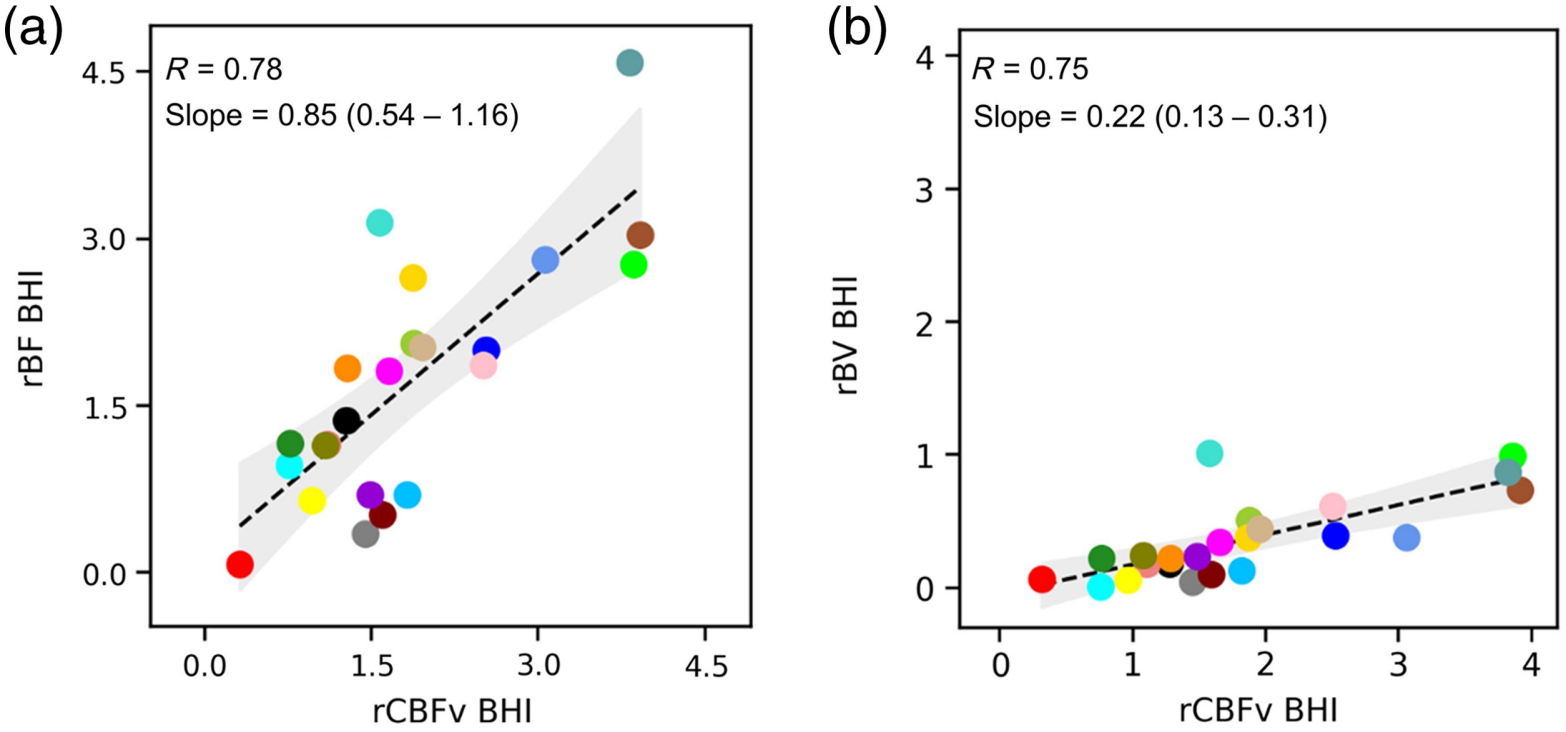
Calculating BHI with optics and TCD: The BHI was calculated for each metric. (a) A scatterplot depicts the BHI based on TCD-derived CBFv (x-axis) and the BHI based on optically derived rBF (y-axis). The correlation coefficient is 0.78. The linear regression coefficient is 0.85 (95% CI: 0.54 to 1.16). (b) A scatterplot depicts the BHI based on TCD-derived CBFv (x-axis) and the BHI based on optically derived rBV (y-axis). The correlation coefficient is 0.75. The linear regression coefficient is 0.22 (95% CI: 0.13 to 0.31). TCD indicates transcranial Doppler. rBF indicates optically measured relative blood flow. rBV indicates optically measured relative blood volume. rCBV indicates TCD measured relative CBFv. BHI indicates breath-hold index.

**Fig. 6:**
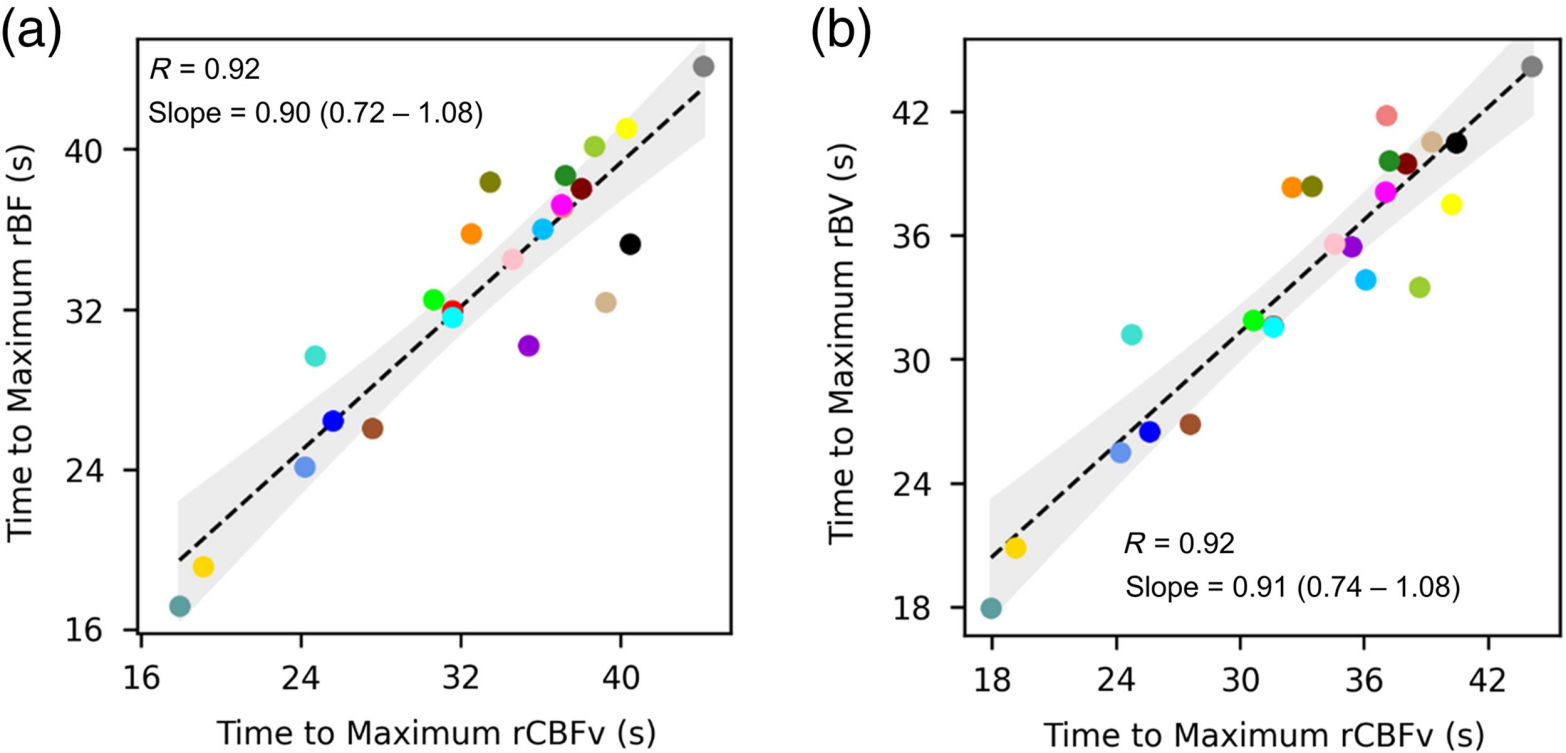
Timing of the cerebral hemodynamic effect: Time (seconds) was calculated from the initiation of the breath-hold to the maximum effect for each metric. (a) A scatterplot depicts the time to maximum effect for rCBFv (x-axis) and for rBF (y-axis). The correlation coefficient is 0.92. The linear regression coefficient is 0.90 (95% CI: 0.72 to 1.08). (b) A scatterplot depicts the time to maximum effect for rCBFv (x-axis) and for rBV (y-axis). The correlation coefficient is 0.92. The linear regression coefficient is 0.91 (95% CI: 0.74 to 1.08). rBF indicates optically measured relative blood flow. rBV indicates optically measured relative blood volume. rCBV indicates relative CBFv. S indicates seconds.

The mean BHI calculated based on optically measured blood flow was 1.71 (±1.07), and the mean BHI calculated based on TCD was 1.85 (±0.99). Good agreement was observed between the BHI calculated by the two modalities [Fig. 5(a)]. The correlation coefficient was 0.78 and the slope of the line of the best fit was 0.85 (95% CI: 0.54 to 1.16). There was also a strong correlation between BHI values calculated based on blood volume and CBFv [Fig. 5(b); R=0.75], but again rBV-based BHI values were expectedly smaller (slope = 0.22; 95% CI: 0.13 to 0.31). The time from breath-hold initiation to maximum cerebral hemodynamic effect was also compared across modalities (Fig. 6), and there was strong agreement between the rBF and rCBFv timing [R=0.92, slope=0.90 (95% CI: 0.72 to 1.08)], as well as the rBV and rCBFv timing [R=0.92, slope=0.91 (95% CI: 0.74 to 1.08)].

**Fig. 7:**
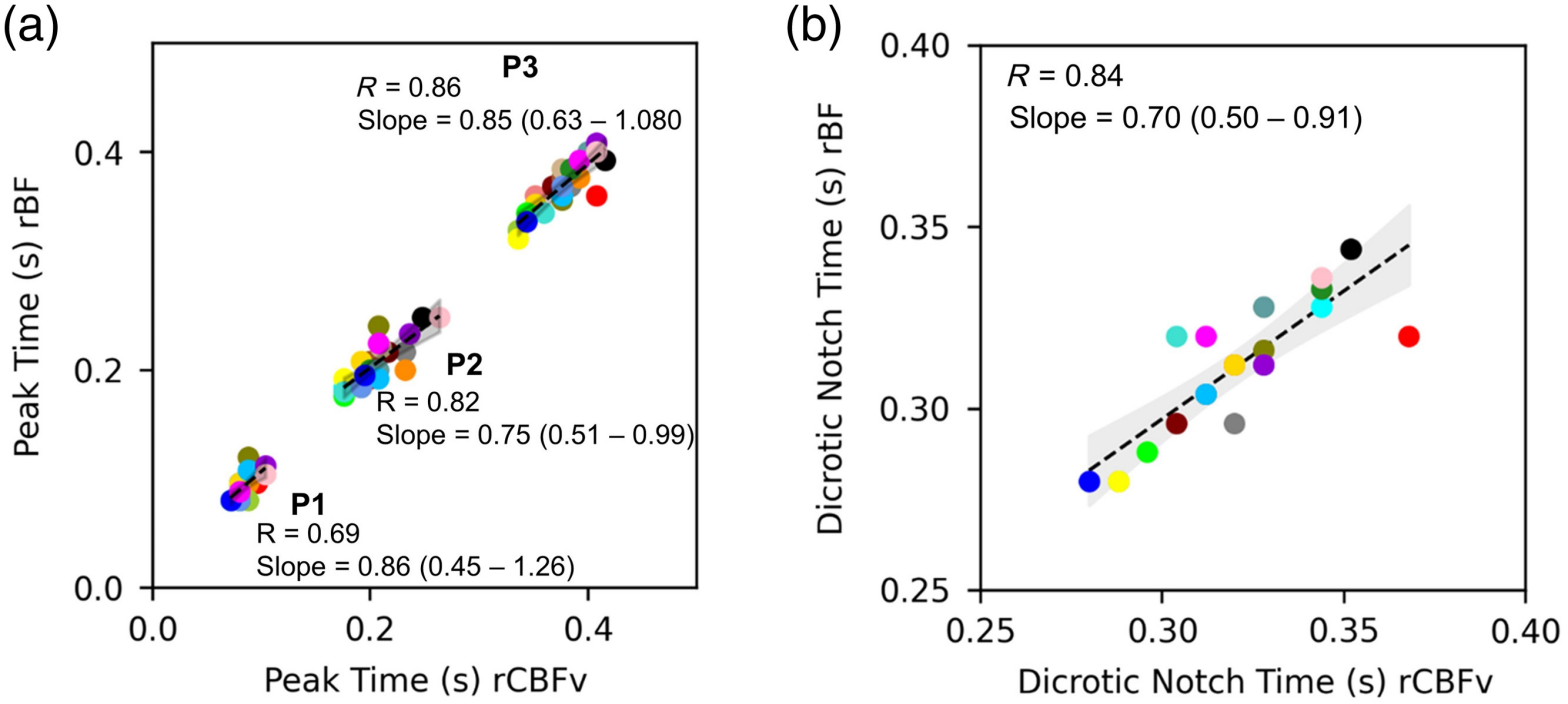
Timing of waveform features: For each subject, waveforms were averaged across the 30 s baseline period. A peak-finding algorithm identified the dicrotic notch, P1, P2, and P3. (a) A scatterplot depicts the timing of each peak based on rCBFv (x-axis) and rBF (y-axis). The correlation coefficient for P1 is 0.69, and the linear regression coefficient is 0.86 (95% CI: 0.63 to 1.08). The correlation coefficient for P2 is 0.82, and the linear regression coefficient is 0.75 (95% CI: 0.51 to 0.99). The correlation coefficient for P3 is 0.86, and the linear regression coefficient is 0.85 (95% CI: 0.45 to 1.26). (b) A scatterplot depicts the timing of the dicrotic notch based on CBFv (x-axis) and rBF (y-axis). The correlation coefficient is 0.84, and the linear regression coefficient is 0.70 (95% CI: 0.50 to 0.91). rBF indicates optically measured relative blood flow. rCBV indicates relative cerebral blood flow velocity. S indicates seconds.

Finally, we compared the timing of morphologic features of the rBF and rCBFv waveform (i.e., P1, P2, P3, and the dichrotic notch; see Fig. 3). There was good agreement between the two modalities with respect to peak timing within the pulse, based on correlation and slope of the best fit line for each peak [Fig. 7(a)]. There was similarly good agreement between the two modalities with respect to the timing of the dicrotic notch within the pulse, with a correlation coefficient of 0.84 and a slope of 0.70 (95% CI: 0.50 to 0.91) for the best fit line [Fig. 7(b)].

**Fig. 8:**
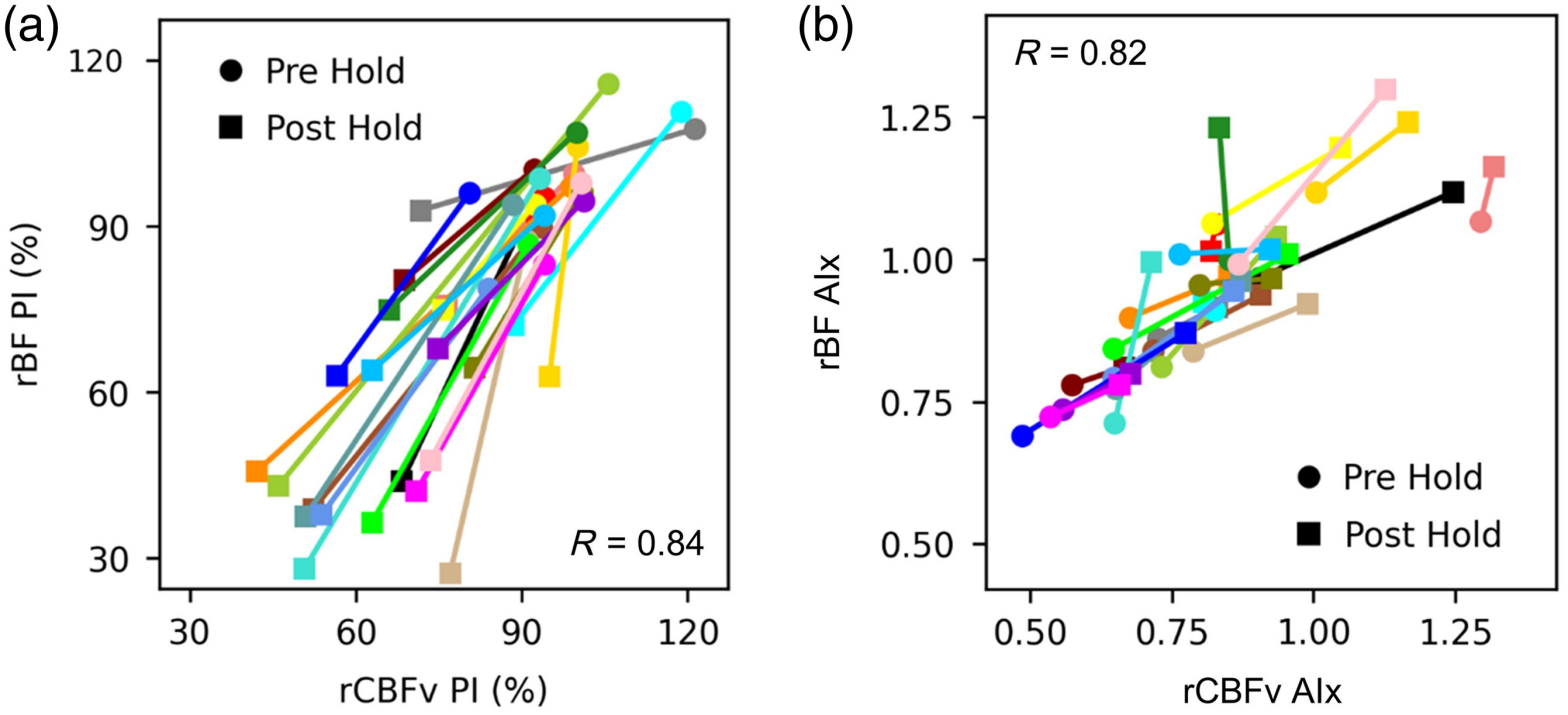
Change in PI and Aix during breath-hold: (a) A scatterplot depicts PI based on rCBFv (x-axis) and rBF (y-axis). Each subject has a data point pre-hold and post-hold. PI is smaller post-hold because the pulse pressure is reduced during hypercapnia. The correlation coefficient is 0.84. (b) A scatterplot depicts the Aix (i.e., P2/P1) based on rCBFv (x-axis) and rBF (y-axis). Each subject has a data point pre-hold and post-hold, and the Aix is larger post-hold that reflects a relative increase in the P2 amplitude. The correlation coefficient is 0.82. rBF indicates optically measured relative blood flow. rCBFv indicates relative cerebral blood flow velocity. PI indicates pulsatility index. AIx indicates augmentation index.

Of further note, an expected reduction in PI was observed during hypercapnia as the pulse width became reduced, and this effect was very strongly correlated between the two modalities [R=0.84; Fig. 8(a)]. Similarly, an expected increase in AIx observed during hypercapnia as the amplitude of P2 increased, and this change was strongly correlated between the two modalities [Fig. 8(b)].

## 4 Discussion

The Openwater Headset is a promising non-invasive optical system that can be leveraged to monitor cerebral hemodynamics at the bedside. This study is the first *in-vivo* validation of the Openwater system. Validation was obtained by comparing changes in optically derived metrics with changes in TCD-metrics during a breath-hold maneuver. Changes in the speckle contrast reflect changes in blood flow and were shown to strongly correlate with TCD at the beat-to-beat level. The BHI measures the overall change in CBF associated with breath-hold and can be calculated with both speckle contrast and TCD with good agreement. Although TCD flow velocity correlated with optical blood volume, as expected, the changes were not proportional because cerebral blood volume pulsatility during the cardiac cycle and blood volume responses to hypercapnia are smaller than the corresponding flow changes.^46^^–^^48^ Importantly, high frequency data collection with the Openwater Headset allows for characterization of blood flow waveform morphology. Openwater and TCD measurement of peak times and clinically useful waveform-based metrics, such as PI and a vascular stiffness index, were very strongly correlated. This initial validation of the Openwater Headset motivates and justifies future validation in larger cohorts and in clinically relevant disease states, such as stroke.

The origin of the Openwater system’s blood flow signal is the speckle intensity fluctuations induced by moving red blood cells. The first technique to exploit speckle intensity fluctuations for non-invasive optical monitoring of deep tissue blood flow in humans was DCS.^49^^,^^50^ DCS uses a long-coherence length laser and homodyne single-photon detection to directly measure the temporal intensity autocorrelation function of the detected light. Correlation diffusion theory is then used to derive a tissue blood flow index from the decay of the autocorrelation function. Several reviews have documented the extensive demonstrations of DCS for CBF monitoring. DCS measurements of the brain, however, are often confounded by low SNR, especially for large source-detector separations. To address this challenge, other methods have been proposed to increase SNR of intensity or electric field autocorrelation function measurements.^20^^,^^51^ These methods include the use of highly parallelized homodyne single photon detection,^52^^–^^56^ heterodyne interferometric detection,^57^^–^^60^ heterodyne holographic detection,^61^^,^^62^ source light with wavelength beyond the water peak,^63^^–^^66^ and high coherence pulsed sources.^67^^–^^73^ The Openwater system does not use high-SNR temporal autocorrelation function measurements to derive blood flow. Instead, it uses speckle contrast to derive blood flow, which is based on an integral of the autocorrelation function. Speckle contrast flow monitoring with low-cost CMOS/CCD has been studied by several groups,^27^^–^^32^^,^^74^^–^^81^ including with wearable probes without fibers, similar to the Openwater system.^33^^–^^35^^,^^81^ Concurrent speckle contrast and DCS monitoring of relative CBF changes have been compared in murine and neonatal swine models,^33^^,^^35^ as well as in human skeletal muscle during cuff-induced forearm ischemia.^27^^,^^29^^,^^30^ Note, however, the source-detector separations used in these comparisons, while appropriate for the respective applications, were comparatively small (i.e., ≤20  mm); larger separations are needed for sensitivity in the adult human brain.^20^ The key difference between the Openwater system and other speckle-contrast-based demonstrations employed to date is the former’s use of very short light pulses (250  μs) to boost SNR (discussed further below). This feature was essential for the high frequency data sampling at 36 mm source-detector separation that enabled discernment of several morphologic features of individual beat-to-beat changes in blood flow. To our knowledge, this discernment of changes in CBF waveform features has not been demonstrated with prior continuous-wave speckle contrast measurements in adult humans. Note, one prior study did demonstrate beat-to-beat changes; it used a rotating chopper wheel outside of the laser system to produce light pulses (>2  ms) longer than those of the Openwater system (250  μs).^32^ We discuss the SNR advantages of the Openwater pulses compared to chopper wheel generated pulses (below).

The correlation between rBF and rCBFv is particularly noteworthy because it was observed not just for steady-state changes during breath-holding, but also for individual beat-to-beat changes. Although the 95% confidence interval of agreement was relatively broad, beat-to-beat values are particularly sensitive to movement artifacts or changes in signal quality over the course of monitoring. Still, the beat-to-beat correlation for each subject was strong. Any subject-level variability that exists is not explained here but could be addressed in a larger cohort with attention to potential contributions from skin pigmentation, age, or skull thickness. In future work, comparison with additional modalities, such as $O^{15}\text{-}\mathrm{PET}$ or ASL-MRI, would provide further validation in a different experimental model.

Change in blood volume was expected to be smaller than the change in flow.^46^^–^^48^ The relationship between flow and volume can be summarized by the central volume principle (CBV = CBF × mean transit time).^82^ With hypercapnia, as flow increases, there is an observed reduction in transit time, which indicates an increase in venous drainage, thereby blunting the increase in volume.^48^ Cerebral blood volume is a key contributor to intracranial pressure, so blunting the increase in blood volume helps to avoid a potentially catastrophic increase in intracranial pressure.^83^ Alternatively, the increase in blood volume may be counterbalanced by displacement of cerebrospinal fluid in order to minimize the effect on intracranial pressure.^84^ In clinical settings, quantifying both rBF and rBV is useful because the combination provides a more thorough characterization of cerebral hemodynamics; e.g., the ratio of flow and volume is informative of transit time and regional perfusion pressure,^48^^,^^85^ which has implications across a range of neurologic disorders, including ischemic stroke, hemorrhagic stroke, subarachnoid hemorrhage, and traumatic brain injury. There are potential systematic errors in quantifying blood volume. Changes in oxygenation could impact intensity without a change in blood volume, but the wavelength of the source laser (785 nm) is very close to the isosbestic point of hemoglobin; therefore, even large changes in oxygenation are expected to result in very small changes in intensity.^86^ Further, at 785 nm, any difference in the overall absorption of hemoglobin should be very small relative to the isosbestic point. Hypercapnia may briefly impact pH, which in turn may affect the hemoglobin absorption spectrum, but this effect is expected to be very small at the end of the breath-hold.^87^

The high frequency data acquisition allows the Openwater Headset to discern several morphologic features of the blood flow waveform. Visualizing the expected peaks and dicrotic notch within the rBF waveform provides an important degree of face validity, and the strong agreement between morphologic features between optical and TCD waveforms is reflective of both construct and content validity. Finally, the correlation between dynamics of PI and AIx provides criterion validity.

The Openwater optical technique described here has sufficient SNR to resolve the CBF waveform because of its use of short pulses of intense light; this approach is unlike continuous-wave SCOS as has previously been reported in the literature.^29^^,^^30^^,^^33^^–^^35^ In a fiber-based SCOS system, Kim et al. observed an improvement in SNR and blood flow waveform detection using a rotating chopper wheel to pulse (>2  ms) the light.^32^ The Openwater system uses shorter pulse lengths (250  μs) that are generated within the laser system, rather than by modulating output laser light. By generating the pulses inside an amplifier section within the laser system rather than outside the laser system, the gain medium is more efficiently used and higher peak power pulses are achieved. The Openwater approach thus enables dynamics to be probed at a shorter time scale, thereby increasing the sensitivity of contrast to small changes in flow and improving waveform detection. The contrast measured using the shorter pulses at large source-detector separation render the Openwater instrument effectively more sensitive to longer photon pathlengths, i.e., pathlengths more biased toward brain than scalp.

In practice, using short intense pulses is technically challenging. Short-pulse high peak power laser operation can lead to chirping, which degrades coherence, and high power laser amplification can result in multiple fluctuating spatial modes that can also reduce SNR. However, our study explicitly shows that these potential complications were not significant (at least for the present measurements at 36 mm source-detector separations on the forehead). The Openwater system is also uniquely designed to include the cameras within the headset (rather than fiber-based headsets), thus ameliorating motion artifacts. The Openwater Headset’s small portable design improves convenience in certain clinical applications. DCS has been used to quantify waveform features^88^^–^^90^ but with lower SNR.^32^

In clinical practice, CBF waveforms are expected to be informative of cerebrovascular resistance, compliance, and intracranial pressure.^90^^–^^92^ TCD-derived CBFv waveform is often interpreted to that end,^93^ but a low-cost user-friendly optical system may have distinct advantages as it evades the need for a trained ultrasonographer and is not limited to patients with adequate temporal acoustic windows. Data in patients with abnormal cerebral hemodynamics would contribute to instrument validation and would help to assess feasibility in an eventual clinical application. For example, a hallmark of acute stroke care is optimization of CBF, but CBF is rarely measured in practice, so there is an opportunity to apply a bedside hemodynamic monitor to facilitate physiology-guided care. As previously described, the ability to measure the blood flow waveform may prove useful, but further study is needed to determine if the optical waveform morphology is informative of clinically relevant pathology, such as elevated intracranial pressure or impaired cerebrovascular compliance.^93^^,^^94^ In acute stroke patients, the TCD waveform may have a role in detection of large vessel occlusions,^95^ but this has not yet been described with biomedical optics.

Despite the encouraging results, this study has several important limitations. Generalizability is limited because of its small numbers and its relatively narrow range of ages and skin pigmentation. Darker skin pigments absorb more light,^96^ so it is critical to demonstrate that the agreement reported here is not pigment-dependent. No test-retest analysis was performed to assess intra-rater reliability because breath holding is often inconstant, but a future study could use a more reproducible change in CBF to evaluate test-retest reliability. Using TCD as the comparator is noteworthy because it provides a measure of CBFv rather than CBF. However, in this experimental model changes are monitored over a very short period of time during which the MCA trunk diameter is expected to remain stable, so relative changes in TCD are reflective of changes in CBF. TCD insonated the MCA trunk, and the Openwater system probes downstream microcirculation. The optical probes were positioned over the lateral aspect of the forehead to monitor the MCA territory, but at this position, there may be some contribution from the anterior cerebral artery. Fortunately, the CBF response to hypercapnia is similar in both the anterior and middle cerebral arteries (i.e., both terminal branches of the internal carotid artery).^97^^–^^99^ Thus, this limitation is not expected to meaningfully impact the correlation between the two modalities. Further, comparison with TCD is reasonable considering it is commonly used to calculate the BHI in clinical practice.^10^^,^^100^^,^^101^ The BHI values reported here are within the range of what is expected in a healthy young cohort but is higher than has been reported in some healthy cohorts.^102^^–^^105^ The difference may be explained by the fact that some groups identify the maximum CBF at the moment the breath-hold ends,^41^^,^^106^ which underestimates the BHI because the maximum CBF is expected to occur a few seconds after the completion of the breath-hold.^100^^,^^106^^,^^107^ Another consideration is the focus on a single breath-hold, rather than averaging multiple breath-holds as is performed by some groups. The hemodynamic response to sequential breath-holds may vary,^102^^,^^108^^,^^109^ and the goal of the current study was to simply induce a large change in CBF to assess agreement between modalities. Thus, a single breath-hold was sufficient. The degree of hypercapnia was not quantified in each subject, which may appear to be a shortcoming, but in actuality the precise change in ${\mathrm{PaCO}}_{2}$ is not relevant to the validation because both modalities were observing the same change in CBF. However, if any subjects had a very small change in CBF, it may have been helpful to know if those subjects had a very small change in ${\mathrm{PaCO}}_{2}$.

## 5 Conclusions

The Openwater system is a promising non-invasive laser speckle-based cerebral hemodynamic monitor. The compact design facilitates portability and the simple user interface emphasizes the potential for future clinical translation. This system’s first *in vivo* validation was demonstrated herein via comparison to TCD. Several data elements were scrutinized to allow for a more robust validation: (1) beat-to-beat changes, (2) BHI, (3) waveform morphology, and (4) dynamics of waveform-based metrics. In total, these analyses are encouraging of future work aimed at validating the Openwater system in disease states, such as stroke, in which a significant need for a practical bedside cerebral hemodynamic monitor exists.

## 6 Appendix A: Instrument Design and Supporting Data

The instrument was designed to maximize sensitivity to small changes in blood flow. That is, for any change in flow, the goal is for the corresponding change in speckle contrast to be as large as possible, without increasing the noise in the system. One key parameter in this optimization is the duration of time over which detected scattered light is hitting the sensor. In the Openwater system, this is determined by the temporal length of the illuminating laser pulse. In traditional speckle contrast experiments, the illuminating laser is continuous (cw), and the shutter-time of the detector is adjusted. In the present approach, since the light is pulsed, the detection “shutter-time” or gating time is the duration of the laser pulse. This scheme maximizes utilization of the illuminating photons, which is particularly valuable when measuring over short time durations; for such cases, the illuminating light intensity often must be very high in order to deliver sufficient light in a short period of time.

In mathematical terms, the optimal laser pulse length maximizes the derivative $\frac{\partial C(F,T)}{\partial F}$ where C is the speckle contrast, T is the pulse length, and F is the flow. This should be computed for physiologically relevant flow rates and optical properties. In general, this derivative will approach zero at the pulse length extremes. For very short pulses, the speckles will not have time to decorrelate and the contrast will remain high over the physiological range of flow rates. For long pulses, during which the speckles have time to decorrelate, the contrast will always be low. In general, for faster flow rates and longer pathlengths (i.e., more scattering events), one expects this change in contrast maximum to occur for shorter laser pulses.

One can readily use the formalism of DCS to model the sensitivity of the speckle contrast to changes in flow.^50^ See Appendix B for details of these calculations. The results are summarized in Fig. S3 in the Supplemental Material. Figure S3(a) in the Supplemental Material depicts the expected speckle contrast as a function of laser pulse length for a wide range of flow rates, i.e., encompassing the rates we have observed in human measurement. As expected, at short pulse widths, the contrast for all flow rates approaches unity, and for longer pulse lengths the contrasts all approach zero. Figure S3(b) in the Supplemental Material depicts the derivative of the contrast with respect to a change in flow as a function of pulse length for the same flow rates. For all flow rates the (negative) peak occurs at ∼200  μs and less. This time is much shorter than the typical exposure times used in LSCI and is a result of the multiple scattering events that occur for each photon in the diffusive transport regime. (Note, these calculations were done with generic optical properties of $\mu_{a}=0.015\;{\mathrm{mm}}^{-1}$ and $\mu_{s}^{\prime }=1.5\;{\mathrm{mm}}^{-1}$ and a source detector separation of 36 mm.) The exact results vary depending on the geometry and optical properties, with higher scattering and longer separations resulting in more scattering events and a shift of the peaks to shorter pulse lengths. However, for physiologically relevant values, the peaks are always well below the >1  ms exposure times used for single scattering LSCI.

**Fig. 9:**
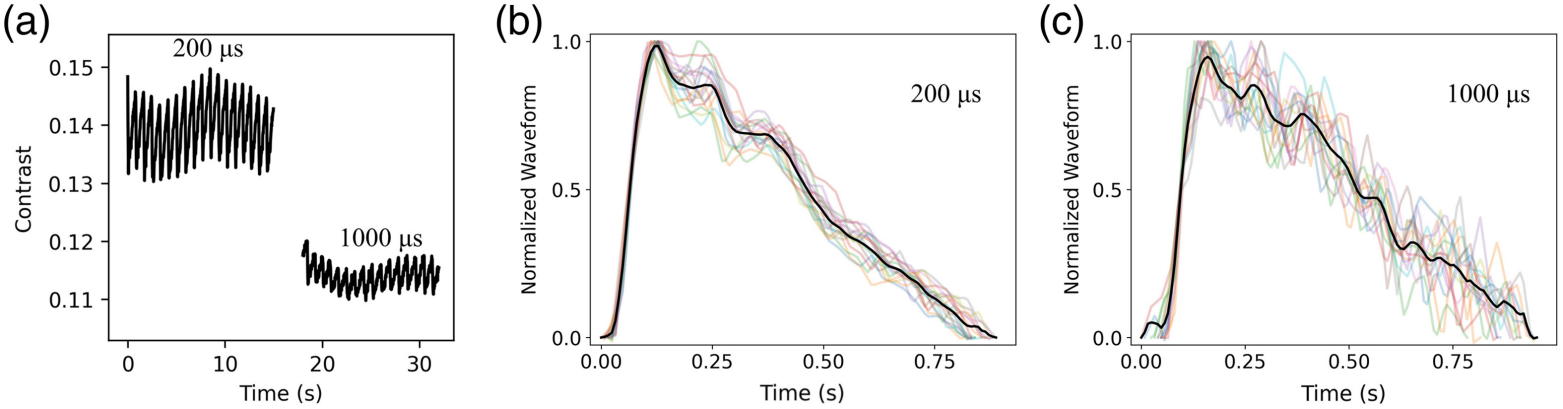
The effect of pulse length on speckle contrast data: While maintaining a constant total energy per pulse (400  μJ), blood flow measurements were compared between two pulse lengths (200 and 1000  μs). (a) The 200  μs pulse width resulted in higher contrast and larger waveform amplitude. For both the (b) 200  μs and (c) 1000  μs pulse widths, each individual heartbeat was readily isolated and waveforms normalized; each waveform was plotted on the same axis. The dark black line represents the average of individual beats. The increased amplitude using 200  μs resulted in a higher SNR.

To demonstrate the effect of pulse length on human measurements, waveforms for one of the subjects were acquired using both 200 and 1000  μs pulse lengths (Fig. 9). For these measurements, the instantaneous power of the 1000  μs pulses was decreased 5× such that the energy per pulse was kept constant for the two pulse lengths at 400  μJ. We found that the shorter pulse width resulted in both a higher speckle contrast and, more importantly, a >3× increase in the amplitude of the waveform [Fig. 9(a)]. The benefit of the increased amplitude translates to improved waveform characteristics with the 200  μs pulses [Figs. 9(b) and 9(c)]. The waveforms derived from the 200  μs pulsed light were more uniform (i.e., were distributed more closely around the average waveform) and had more favorable signal compared to noise.

The same two laser pulse widths were also applied to a static optical phantom with similar optical properties as human tissue. As expected, the resulting contrast values were much higher with C=0.28±0.00051 at 200  μs, and C=0.29±0.00065 at 1000  μs. In both cases, the variation in the measured contrast was about 0.2% of the mean value. Given that phantom was static, ideally the measured speckle contrast should be the same for both pulse lengths. The slight decrease (∼3%) in speckle contrast between the 1000  μs pulses and the 200  μs pulses is attributed to a slight decrease in laser performance resulting from the combination of the 5× pulse length decrease, and the 5× instantaneous power increase.

## 7 Appendix B: Analytic Expressions for Speckle Contrast and Its Sensitivity to Changes in Flow

In this appendix, we derive analytic expressions for the speckle contrast C, and the derivative of the speckle contrast as a function of blood flow $\frac{\partial C}{\partial (\alpha D_{b})}$. These expressions are used in the laser pulse length analysis of Appendix A. They also provide a straightforward and rapid way to predict the effect of optical properties and source detector separations on the speckle contrast.

Working within the framework of DCS, we use a homogeneous semi-infinite model to calculate the electric field temporal autocorrelation function:

$$G_{1}(\tau )=\frac{\exp\{-K(\tau )\rho \}}{\rho^{2}}.$$

Assuming that the moving scatterers (e.g., red blood cells) undergo Brownian motion, and all other scatterers are motionless we have:

$$K^{2}(\tau )=3\mu_{a}\mu_{s}^{\prime }+\mu_{s}^{\prime 2}k_{0}^{2}\alpha 6D_{b}\tau .$$

Here, ρ is the source-detector separation of point-like source/detector on the surface, $\mu_{s}^{\prime }$ is the reduced scattering coefficient, $\mu_{a}$ is the absorption coefficient, $k_{0}$ is the light wavenumber, α is the fraction of moving scatterers, $D_{b}$ is the effective Brownian diffusion coefficient of the moving scatterers, and τ is the delay time. In this model, the blood flow is defined as $\alpha D_{b}$. This term is the product of the fraction of scatterers that are moving (α) and rate, $D_{b}$, at which these scatterers diffuse through the tissue.^50^ In order to calculate the speckle contrast, we integrate the normalized electric field autocorrelation function:

$$g_{1}(\tau )=\exp\{(K(0)-K(\tau ))\rho \},$$

according to

$$C=\frac{2}{T}\int_{0}^{T}(1-\frac{\tau }{T}){\left|g_{1}(\tau )\right|}^{2}d\tau .$$

Here, T is the light exposure time (laser pulse length in the present case), and C is the speckle contrast. This integral can be solved analytically. To do so, it is easier to proceed if we define the following parameters

$$a=3\mu_{a}\mu_{s}^{\prime }{\left(2\rho \right)}^{2}\;\text{and}\;b=\mu_{s}^{\prime 2}k_{0}^{2}\alpha 6D_{b}(2\rho {)}^{2}.$$

Using this notation, the solution is

$$C=\frac{2}{bT}{\left\{e^{\sqrt{a}-\sqrt{a+bT}}[2(a+bT)+6\sqrt{a+bT}+6]-[2a+6\sqrt{a}+6]+bT(\sqrt{a}+1)\right\}}^{1/2}.$$

From this equation, it is straight forward to compute the derivative of the contrast with respe to flow (i.e., $\alpha D_{b}$). It is

$$\frac{\partial C}{\partial (\alpha D_{b})}=\frac{1}{\alpha D_{b}}\{\frac{2}{bTC}[(\sqrt{a}+1)-(\sqrt{a+bT}+1)]e^{\sqrt{a}-\sqrt{a+bT}}-C\}.$$

Note, these equations represent the case of polarized light and a pixel size much smaller than the spatial coherence area on the sensor. In practice, all values of the contrast are scaled by a constant less than one, often represented by $\sqrt{\beta }$. in the speckle contrast imaging literature.

## Supplementary Material





## Data Availability

The data that support the reported findings are available from the corresponding author upon reasonable request.
