# Supplementary material for: Validation of the Openwater wearable optical system: cerebral hemodynamic monitoring during a breath-hold maneuver
Source: Neurophotonics. 2024 Mar 8;11(1):015008. doi: 10.1117/1.NPh.11.1.015008 (PMC10923543; doi:10.1117/1.NPh.11.1.015008)
Supplement: Supplementary file 1 [file NPh_011_015008_SD001.pdf]

Supplementary Material

Figure S1. Schematic of instrumentation

Figure S2. Comparing left and right optical metrics during a breath-hold maneuver

Figure S3. Sensitivity of speckle contrast to flow across a range of laser pulse lengths

Figure S1.

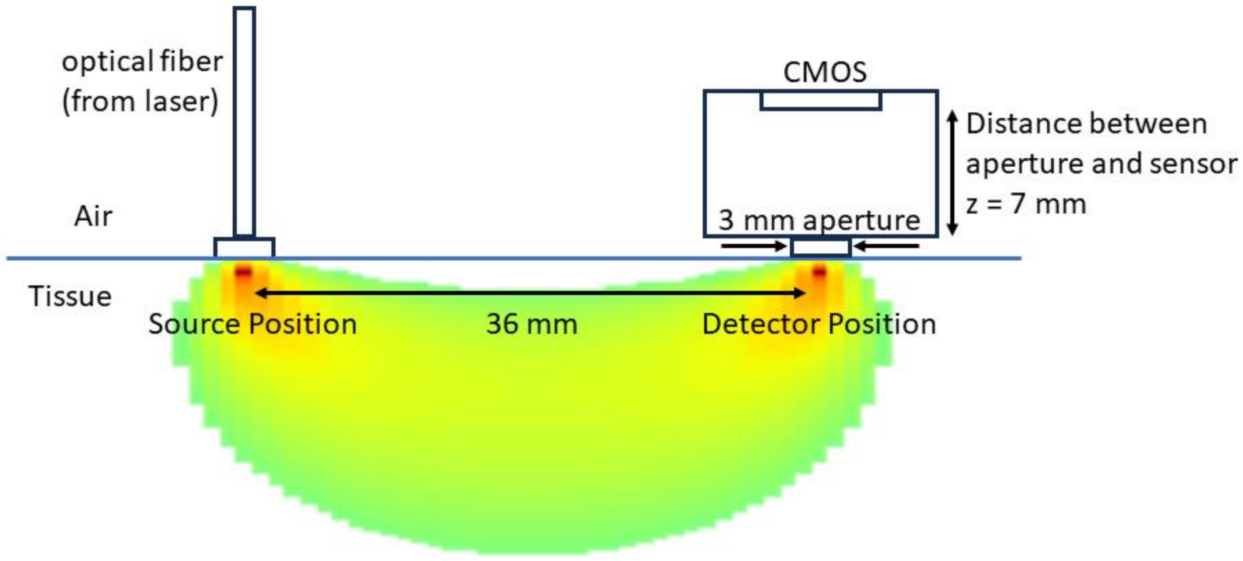

Figure S1. Schematic of instrumentation: The source and detector are positioned at a distance of 36 mm. Within the custom camera, a CMOS sensor is recessed 7 mm from the aperture.

Figure S2.

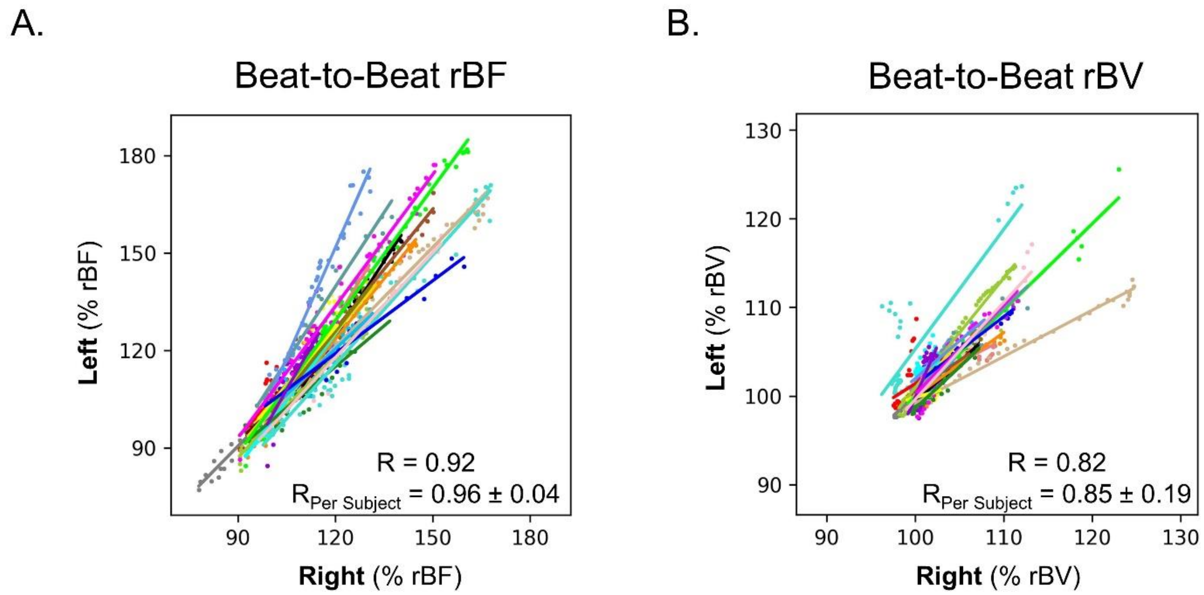

Figure S2. Comparing left and right optical metrics during a breath-hold maneuver: All data are normalized to the 30-second period preceding the breath hold. Beat-to-beat mean values are calculated for each metric from the start of the breath hold through 5 seconds after the completion of the breath hold. Each color represents a different subject. (A) A scatterplot depicts the beat-to-beat mean rBF from the right (x-axis) and left probes (y-axis). The overall correlation coefficient is 0.92. The average correlation coefficient (when calculated for each subject individually) is 0.96 (+/- 0.04). (B) A scatterplot depicts the beat-to-beat mean rBV from the right (x-axis) and left probes (y-axis). The overall correlation coefficient is 0.82. The average correlation coefficient (when calculated for each subject individually) is 0.85 (+/- 0.19). rBF indicates optically measured relative blood flow. rBV indicates optically measured relative blood volume.

Figure S3.

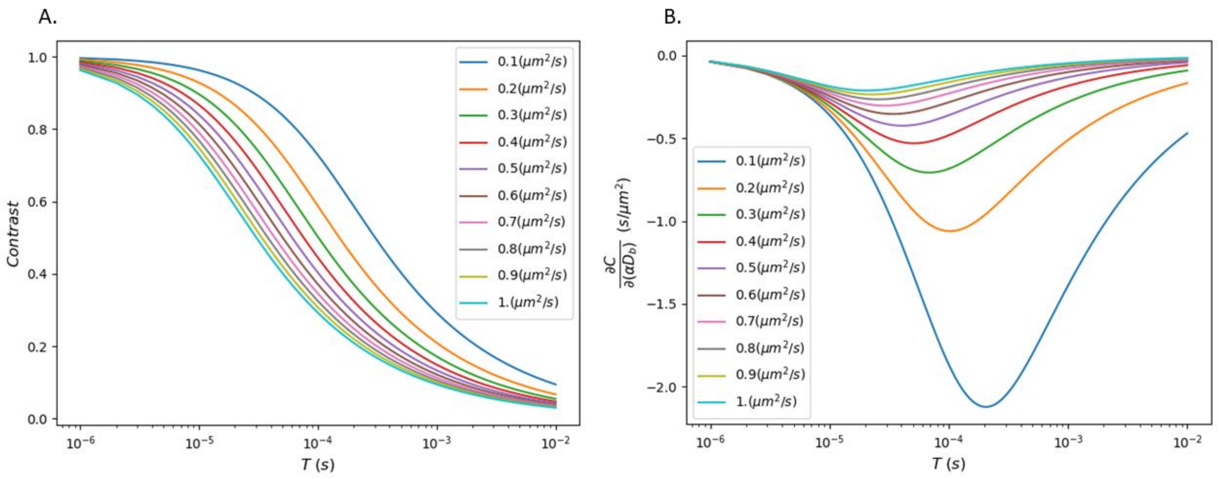

Figure S3. Sensitivity of speckle contrast to flow across a range of laser pulse lengths: For a wide range of flow rates αD_b, (A) the speckle contrast and (B) the derivative of the speckle contrast with respect to flow are plotted as a function of pulse length. The maximum sensitivity of contrast to changes in flow occurs for pulses of 200 μs and below.
